# Epidemiologic Features of 135 Patients With Coronavirus Disease (COVID-19) in Tianjin, China

**DOI:** 10.1017/dmp.2020.63

**Published:** 2020-04-01

**Authors:** Chunxia Cao, Yue Li, Shuyu Liu, Haojun Fan, Liangchen Hao

**Affiliations:** 1 Institute of Disaster Medicine, Tianjin University, Tianjin, China; 2 Emergency Management Office, Tianjin Health Commission, Tianjin, China

**Keywords:** coronavirus disease, epidemiology, novel coronavirus

## Abstract

**Objective:**

This study describes the epidemiologic features of an outbreak of the coronavirus disease (COVID-19) in Tianjin caused by a novel coronavirus and provides the scientific basis for prevention and control measures.

**Methods:**

Data from COVID-19 cases were collected from daily notifications given to the National Health Commission of the People’s Republic of China and Tianjin Health Committee. All of the data were analyzed with SPSS, version 24.0 software (IBM Corp, Armonk, NY).

**Results:**

As of February 24, 2020, there have been 135 confirmed cases, 3 deaths, and 87 recoveries in Tianjin, China. The incidence of COVID-19 was 8.65/1 000 000 with a 2.22% case fatality rate. Regarding geographic distribution, the incidence was 8.82 per 1 000 000 in urban areas and 8.00 per 1 000 000 in suburbs. During the early stage of the epidemic, most cases came from urban areas and in patients with a history of sojourning in Hubei Province. The majority of patients were 31–70 years old (75.97%). A familial clustering was the most important characteristic of COVID-19 (accounting for 74.81%).

**Conclusions:**

Current information suggests that people are generally susceptible to COVID-19, which has shown a familial clustering in Tianjin.

## INTRODUCTION

The 2019 novel coronavirus is the coronavirus identified as the cause of an outbreak of the coronavirus disease (COVID-19) first detected in the city of Wuhan, Hubei Province, China, at the end of 2019.^1^ Early on, many of the patients in the Wuhan outbreak reported some contact with a large seafood and animal wholesale market, suggesting animal-to-person spread. However, a growing number of patients have reported no such exposure to animal markets, suggesting a possible person-to-person spread.^2,3^ COVID-19 was imported to Tianjin on January 21, 2020. Tianjin Municipal authorities took aggressive measures to improve detection, isolate COVID-19 patients, and trace contacts to minimize opportunities for further transmission in community and institutional settings.^4^ We summarize some epidemiologic features of the outbreak of COVID-19 in Tianjin during 2020, and we attempt to explore prevention and control measures.

## METHODS

### Setting

Tianjin municipality has an estimated population of 15.6 million and includes 11 districts and 5 counties. We defined the districts of Heping, Hexi, Hedong, Nankai, Hebei, and Hongqiao as urban areas. These areas have a total population of 5.1 million. We defined the districts and counties of Dongli, Xiqing, Jinnan, Beichen, Baodi, Wuqing, Ninghe, Jinghai, Jixian, and Binhai New Area (including Tanggu, Dagang, and Hangu) as suburban areas. Together, they have a population of 10.5 million.

### Source of Data and Diagnostic Criteria

All of the data of COVID-19 cases were collected from daily notifications given to the National Health Commission of the People’s Republic of China and Tianjin Health Committee.^4,5^ The cases were diagnosed according to the diagnostic and treatment guidelines for COVID-19 issued by the Chinese National Health Committee (Version 3–5).The hospital admission date and the severity of COVID-19 cases were recorded. There were 135 clinically diagnosed COVID-19 cases (admission data from January 21 to February 24, 2020) in Haihe Hospital of Tianjin. Haihe Hospital of Tianjin is the only designated hospital for the hospitalization of patients with COVID-19 in Tianjin. Clinically, the disease is divided into 4 types of cases: mild, ordinary, severe, and critical. Mild cases are defined as the cases with mild clinical symptoms and no pneumonia manifested through imaging results. Ordinary cases have fever, respiratory symptoms, and others symptoms of viral pneumonia manifested through imaging results. Severe cases meet any 1 of the following: (1) respiratory distress: RR ≥ 30 times/min; (2) fingertip oxygen saturation ≤ 93% under resting state; and (3) arterial partial pressure of oxygen (PaO_2_)/fraction of inspiration O_2_ (FiO_2_), P/F ≤ 300 mmHg (1 mmHg = 0.133 kPa). Critical cases meet any 1 of the following: (1) respiratory failure and an artificial airway required for invasive mechanical ventilation; (2) shock; and (3) combining failure of organs, which require intensive care unit monitoring and treatment.

### Statistical Analysis

A total of 135 valid data were entered into a dataset file with Microsoft Excel 2010 and analyzed with SPSS, version 24.0 (IBM Corp, Armonk, NY). The incidence, mortality, and case fatality rates were calculated, and other distribution characteristics are described here.

## RESULTS

### COVID-19 Incidence, Mortality, Case Fatality Rates, and Severity in Tianjin, 2020

There was a total of 135 confirmed cases of COVID-19 in residents whose addresses were reported as being in Tianjin as of February 24, 2020. There were 45 in urban areas, 84 in suburban areas, and 6 from other provinces. There were 87 cured cases (64.44%), 32 common cases (23.70%), 11 severe cases (8.15%), 2 critical cases (1.48%), and 3 fatal cases (2.22%). The incidence and mortality rate were 8.65 per 1 000 000 in urban areas and 0.19 per 1 000 000 in rural areas. The fatality rate in Tianjin was 2.22%. The incidence in urban and suburban areas was 8.82 per 1 000 000 and 8.00 per 1 000 000, respectively. Nine mixed medical assistance teams were set up by Haihe Hospital of Tianjin, with a total of 153 clinicians and 369 nurses.

### Date of Disease Onset

The first 4 cases in Tianjin were identified on January 21. The outbreak peak appeared on January 27 and February 6, 2020, in urban and suburban areas, respectively. In the first 8 days of the outbreak, urban cases accounted for the majority of daily new cases (23; 82.14%). As of February 1, suburban cases have made up the majority (79; 78.22%). In the first 8 days of the outbreak, severe cases accounted for the majority of daily new cases (17; 70.83%). As of February 24, recovered cases have made up the majority (87; 64.44%).

### Distribution of COVID-19 Cases by Age

The youngest case in Tianjin was 9 years old and the oldest was 90 years old. The mean age was 48.87 years (48.87 ± 16.91 years). The age of COVID-19 cases in urban areas ranged from 19 to 90 years, averaging 51.89 years (51.89 ± 15.79 years), whereas the age of COVID-19 cases in suburban areas ranged from 9 to 90 years, averaging 47.89 years (47.89 ± 17.12). There was no significant difference in age between infected individuals from the 2 areas (*t* = 1.298, *P* = 0.197). The distribution of COVID-19 cases by age is shown in Table 1.

**TABLE 1:** Distribution of COVID-19 Cases in Tianjin

Variable	Urban Area		Suburban Area		Total^a^		t or χ^2^, *P*
	No. of cases	%	No. of cases	%	No. of cases	%	
Age							
1–9	0	0.00	2	2.50	2	1.55	1.298, 0.197
10–19	2	4.44	0	0.00	2	1.55	
20–29	3	6.67	11	13.75	14	10.85	
30–39	6	13.33	17	21.25	23	17.83	
40–49	11	24.44	17	21.25	28	21.71	
50–59	11	24.44	14	17.50	25	19.38	
60–69	6	13.33	16	20.00	22	17.05	
70–79	5	11.11	5	6.25	10	7.75	
80–90	1	2.22	2	2.50	3	2.33	
Gender							
Male	29	64.44	38	45.20	67	51.94	4.330, 0.037
Female	16	35.56	46	54.80	62	48.06	
Cluster							
Family	23^b^	71.87	51^b^	66.23	74^b^	67.89	0.330, 0.566
Social^c^	9^b^	28.13	26^b^	33.77	35^b^	32.11	
Contact History							
Yes	14	31.10	6	7.14	20	15.50	13.850, 0.001
No	31	68.90	78	92.86	109	84.50	

a Six cases from other provinces were excluded.

b Some COVID-19 cases have to do with both family clusters and social clusters.

c The social place cluster includes workplace and public place cluster.

### Distribution of COVID-19 Cases by Gender

Of all of the COVID-19 cases in Tianjin, 72 were male and 63 were female, accounting for 53.33% and 46.67%, respectively. There were 29 (64.44%) male cases and 16 (35.56%) female cases in the urban areas, and 38 (45.20%) male cases and 46 (54.80%) female cases in suburban areas. The gender difference was found in the cases between urban and suburban areas (χ^2^ = 5.398, *P* = 0.020). There were more male cases in urban areas and more female cases in suburban areas. The distribution of COVID-19 cases by gender is shown in Table 1.

### Familial Clustering of COVID-19 Patients

**FIGURE 1:**
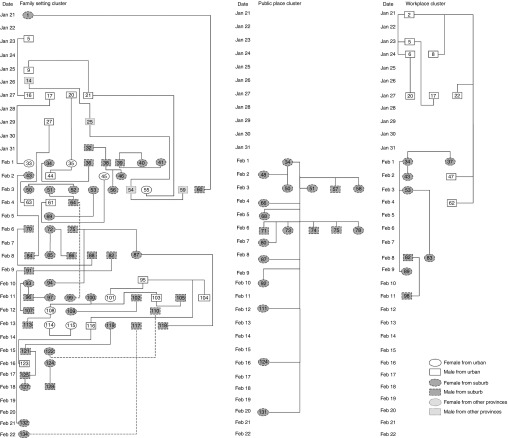
Familial Clustering of COVID-19 Patients. (The Numbers in Circles or Boxes Indicate the Codes of Confirmed Cases.)

Of the total COVID-19 cases, 101 (74.81%) cases belonged to family or social setting (including workplace and public place) clusters. There were 78 (57.78%) cases of family setting clusters, 17 (12.59%) cases of workplace clusters, and 18 (13.33%) cases of public place clusters. Table 1 shows 23 (71.87%) family setting clusters and 9 (28.13%) social setting clusters in the urban areas, and 51 (66.23%) family setting clusters and 26 (33.77%) social setting clusters in the suburban areas. No difference was found among the cases between family setting and social setting clusters (χ^2^ = 0.330, *P* = 0.566). Figure 1 shows the familial clustering of COVID-19 patients.

### History of Sojourning in Hubei Province

Of the 135 COVID-19 cases in Tianjin, 26 (29.55%) came into contact with the epidemic area before the onset of symptoms, but 109 (80.74%) had no history of contact with the epidemic area before the onset. During the first 10 days of the outbreak, cases with a history of sojourning in Hubei Province accounted for the majority of new cases every day (19; 61.29%). As of January 31, COVID-19 cases with no history of sojourning in Hubei began to account for the majority of the new cases. Even on January 31, February 1, and February 5–24, all of the new case patients daily had not been to Hubei Province. Among urban residents, there were 14 (31.10%) COVID-19 cases of people who had visited Hubei Province and 31 (68.90%) COVID-19 cases of people who had not. Among suburban residents, there were 6 (7.14%) COVID-19 cases of people who had visited Hubei Province and 78 (92.86%) COVID-19 cases of people who had not. The difference between the 2 areas was significant (χ^2^ = 12.850, *P* = 0.001) (see Table 1).

## DISCUSSION

Tianjin municipality has an estimated population of 15.6 million across its 11 districts and 5 counties. The first case of COVID-19 was detected in Tianjin on January 21, 2020. The COVID-19 outbreak struck not only the urban areas, but also the suburbs. All of the 14 districts and counties were affected by COVID-19. The number of people living permanently in Tianjin was used as the denominator to compute the incidence of COVID-19. The familial clustering was found to be the most important characteristic of COVID-19. Patients with familial clustering accounted for 74.81% of cases, and there was no difference in clustering between urban and suburban areas. Infection was not found to be related to contact with animals. It has been preliminarily concluded that the source of infection is human.^6,7^

The attack rate in urban areas was significantly higher than the attack rate in suburban areas. However, of the 135 COVID-19 patients, 87 cases were common, accounting for 64.44%. The condition was relatively serious, and the case-fatality rate was 2.22%. The youngest case was 9 years old and the oldest was 90. The mean age was 48.87. With regard to the gender distribution of COVID-19 cases, 72 were male and 63 were female. The distribution by age and gender in urban and suburban areas showed no significant difference. The distribution by gender in urban and suburban areas showed a significant difference. There was a public place cluster in an urban area, and a total of 9 infected subjects were males, accounting for 31.03% of the male patients in the urban area. The 24 cluster cases in the suburbs were all linked to a local department store, 20 of which were female, accounting for 43.48% of the male patients in the urban area. The result may attribute to more male in the urban areas and more women suffering from the disease in suburban areas. These results indicate that the population is generally susceptible.

Although 19.26% of COVID-19 cases in Tianjin were reported in people who had been in contact with the epidemic area before the onset of symptoms, cases with a history of visiting Hubei Province accounted for the majority of new cases for each of the first 10 days of the outbreak. The proportion of case patients both in urban and suburban areas who had a history of contact with COVID-19 patients or epidemic areas was higher than among those who had such history, and the difference was found to be significant. Over time, the new COVID-19 cases emerged among people with no history of sojourning in Hubei Province. This raised the possibility that person-to-person transmission was the main form of transmission. Tianjin Municipal authorities took aggressive measures to protect public health and prevent the spread of COVID-19.^8^ There were no new confirmed cases in Tianjin for 2 consecutive days (February 23 and February 24, 2020).

## CONCLUSION

All of the COVID-19 case patients were admitted to hospitals in urban and suburban areas, indicating that the hospitals in Tianjin had sufficient capacity to admit and treat all of the cases efficiently at that time. These results indicated that COVID-19 was an infectious disease with a familial clustering and there was a high incidence of crowd susceptibility in Tianjin.
